# Understanding the Enhanced Osmotic Energy Conversion in Heterogeneous Membranes Using Engineered Branched Alumina Nanochannel Membranes

**DOI:** 10.1002/smsc.202300167

**Published:** 2023-11-27

**Authors:** Yen-Shao Su, Amalia Rizki Fauziah, Chung-Yi Wong, Ting-Yi Huang, Li-Hsien Yeh

**Affiliations:** ^1^ Department of Chemical Engineering National Taiwan University of Science and Technology Taipei 10607 Taiwan; ^2^ Advanced Manufacturing Research Center National Taiwan University of Science and Technology Taipei 10607 Taiwan

**Keywords:** ion transports, ionic current rectifications, nanofluidics, rectified membranes, salinity gradient powers

## Abstract

Heterogeneous membranes with two composite layers of distinct pore sizes have emerged as promising candidates for efficient osmotic energy extraction from salinity gradients. Previous studies about heterogeneous membranes report a strong link between the induced diode‐like rectification property and improved osmotic energy conversion performance. Nevertheless, the inherently large interfacial resistance of heterogeneous membranes may offset such enhancement. To further understand the osmotic energy conversion behavior in heterogeneous membranes, a series of the branched alumina nanochannel (BAN) membranes consisting of large stem channels interconnected with small branched channels are designed. The interconnected and orderly aligned structure of the stem and branched channels ensures high effective driving force and ion selectivity while reducing interfacial resistance at the same time. Experimental and simulation results confirm the rectification effect induced by the asymmetric pores of BAN, which can achieve an osmotic power ≈130% higher than that of the conventional cylindrical nanochannel membranes. Additionally, a power density as high as 5.42 W m^−2^ is obtained by mixing seawater and river water, surpassing most of the existing heterogeneous membranes and the commercial benchmark. The BAN membrane proposed in this work provides a promising platform for the development of highly efficient osmotic energy harvesting devices.

## Graphical abstract

The branched alumina nanochannel membrane is utilized to understand the improved osmotic energy performance in conventional heterogeneous membranes. Its interconnected and orderly aligned pore structure of stem and branched channels not only induce ionic rectification, but also promote the effective driving force and demote the interfacial resistance, thus rendering it as a high‐performance platform for osmotic energy harvesting.

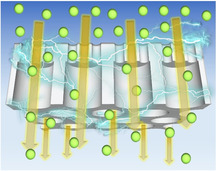

## 1 Introduction

Osmotic energy, or blue energy, is a type of renewable energy that can be extracted from the salinity gradient between seawater and river water.^[^ ^1^, ^2^ ^]^ Due to the accessibility and abundance of ocean water, osmotic energy has emerged as a potential substitute for the existing nonrenewable energy sources such as fossil fuels, for which the demand far exceeds its supply. On top of that, the cleaner process of osmotic energy harvesting can be utilized to alleviate the tremendous environmental threats caused by the excessive exploitation of the fossil fuels.^[^ ^3^ ^]^

As the scavenging of chemical potential energy between high‐ and low‐salinity solutions is dictated by the mass transport of the charged ions, a commonly adopted tool for blue energy generation is using ion‐selective membranes consisting of charged nanoscaled channels, which could promote directional and even enhanced ion transport.^[^ ^4^, ^5^, ^6^, ^7^ ^]^ In a charged nanochannel, when the thickness of the electrical double layer (EDL) is comparable to the channel size, the majority of counterions are able to travel through the channels, thus ensuring a sufficiently high ion selectivity.^[^ ^8^ ^]^ Nevertheless, the EDL overlapping effect in the same nanochannels will at the same time induce ion concentration polarization (ICP),^[^ ^9^, ^10^, ^11^ ^]^ which in turn yields a noticeable enrichment of total ionic concentration near the nanochannel entrance, thereby reducing the driving force for osmotic ion transport.^[^ ^12^, ^13^ ^]^

Heterogeneous or composite membranes are among the most popular choices for osmotic energy harvesting from salinity gradients due to their tunable membrane properties and variable cross‐membrane channel sizes for enhanced ion selectivity, permeability, and hence osmotic power output.^[^ ^14^, ^15^, ^16^, ^17^, ^18^, ^19^, ^20^, ^21^, ^22^, ^23^, ^24^, ^25^, ^26^, ^27^, ^28^, ^29^, ^30^ ^]^ Anodized aluminum oxide (AAO) with aligned nanochannels^[^ ^31^ ^]^ is a common material for constructing nanoporous substrates for these membranes due to its highly ordered and tunable pore structure and geometry.^[^ ^19^, ^20^, ^21^, ^22^, ^23^, ^24^, ^25^, ^26^, ^27^, ^28^, ^29^, ^30^ ^]^ Moreover, the enhanced osmotic energy conversion performance with the heterogeneous membranes is typically linked to the induced diode‐like ion current rectification (ICR) property,^[^ ^32^ ^]^ which is able to provide unidirectional ion transport with amplified conductance.^[^ ^33^, ^34^ ^]^ However, the understanding of the enhanced osmotic power in the heterogeneous membrane with distinct pore sizes is still at the infancy stage.

**Figure 1:**
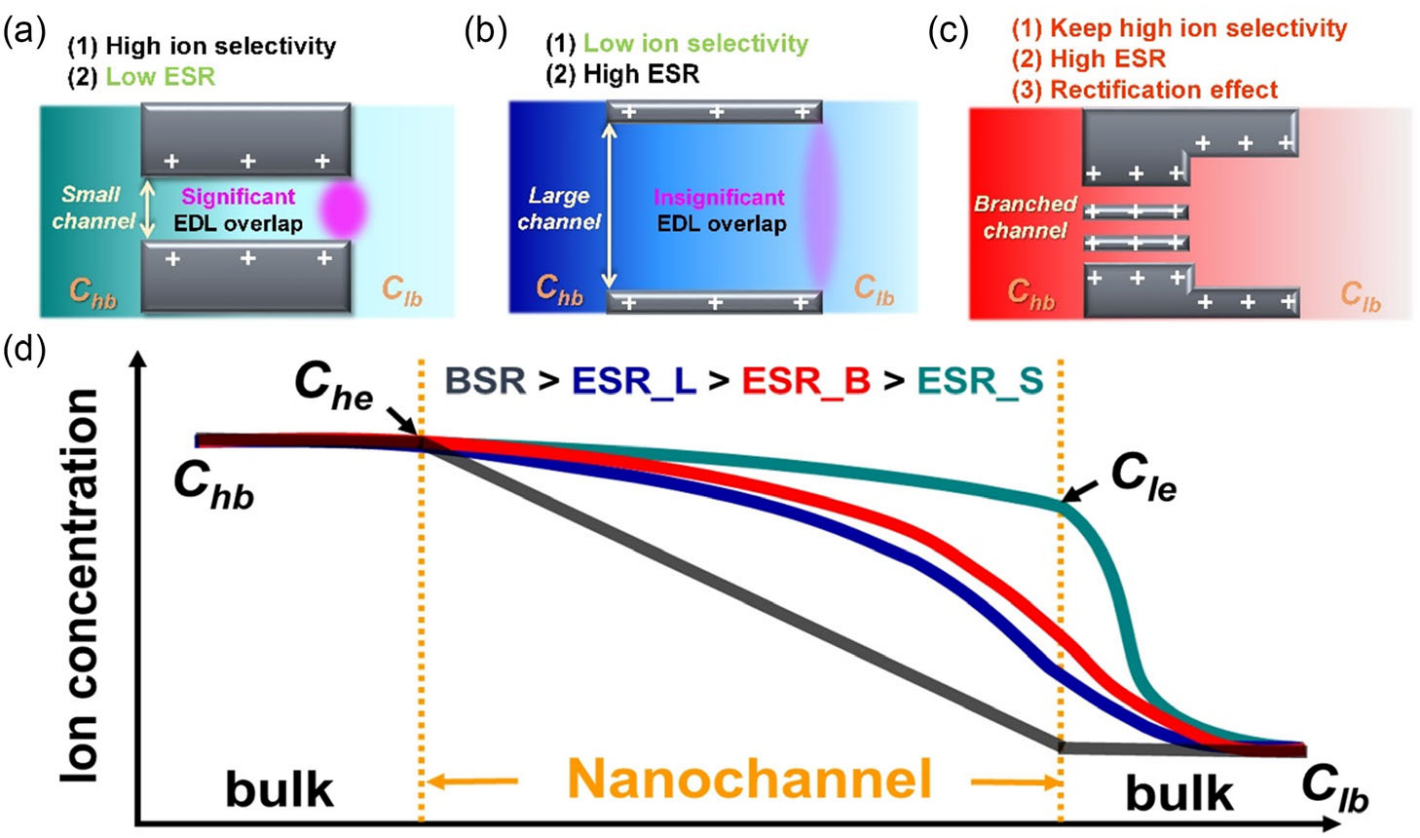
Proposed mechanisms of osmotic ion transport in the a) small nanochannel, b) large nanochannel, and c) branched nanochannel systems. d) Proposed axial variations of total ionic concentration profiles in the small (green curve), large (blue curve), and branched (red curve) nanochannels. BSR denotes the bulk salinity ratio applied through a nanochannel, which is estimated as *C*_hb_/*C*_lb_. *C*_hb_ and *C*_lb_ denote the bulk salt concentrations applied in the high‐concentration and low‐concentration reservoirs, respectively. ESR denotes the ESR across a nanochannel and can be estimated as *C*_he_/*C*_le_. *C*_he_ and *C*_le_ denote the effective salt concentrations at the high‐concentration and low‐concentration entrances of a nanochannel, respectively. Due to the ICP effect, the ESR in ion‐selective channel membranes is smaller than the BSR. It is expected that ESR values in the small (ESR_S), large (ESR_L), and branched (ESR_B) nanochannel systems are ranked in the order: ESR_L > ESR_B >> ESR_S.

In this article, we tackle a question of why the heterogeneous membranes with distinct two pore sizes can enhance the nanofluidic osmotic energy harvesting performance by utilizing the engineered branched alumina nanochannel (BAN) membrane. The fabricated BAN membrane is composed of large stem channels interconnected with branched channels of smaller pore size (Figure S1, Supporting Information). It is known that the conventional small nanochannel membrane is characterized by strong ion selectivity, but significant EDL overlapping effect in the channel leads to apparent ICP effect and thus small effective salinity ratio (ESR) across the membrane,^[^ ^13^ ^]^ which is the actual driving force for osmotic power generation (**Figure** **1**a). On the other hand, the large nanochannel membrane is characterized by weak ion selectivity even though the insignificant ICP effect can promote the ESR for osmotic power (Figure 1b). It is expected and will be shown later that the BAN membrane can retain the two merits of small and large channel membranes (Figure 1c). Its large stem channels can achieve a high ESR by undermining the ICP effect, while the small branched channels can maintain ion selectivity through the overlapping of EDL (Figure 1c,d). Moreover, the broken symmetry in pore sizes in the BAN membrane could induce ICR property. Since ion selectivity, ESR, and ICR can affect the performance of osmotic energy conversion, the BAN membrane is proposed for high‐performance osmotic energy generation. In this work, we also perform the simulations based on the multi‐ion Poisson–Nernst–Planck and Navier–Stokes (PNP–NS) models^[^ ^12^, ^35^ ^]^ (see details in the Experimental section and Supporting Information), which show good agreement with our assumptions depicted in Figure 1. Finally, we explored the optimal design for the BAN membrane, which can achieve a high‐performance power density of up to 5.42 W m^−2^ by mixing artificial seawater and river water, exceeding the commercial benchmark (5 W m^−2^).^[^ ^6^ ^]^

## 2 Results and Discussion

### 2.1 Fabrication of BAN Membranes

**Figure 2:**
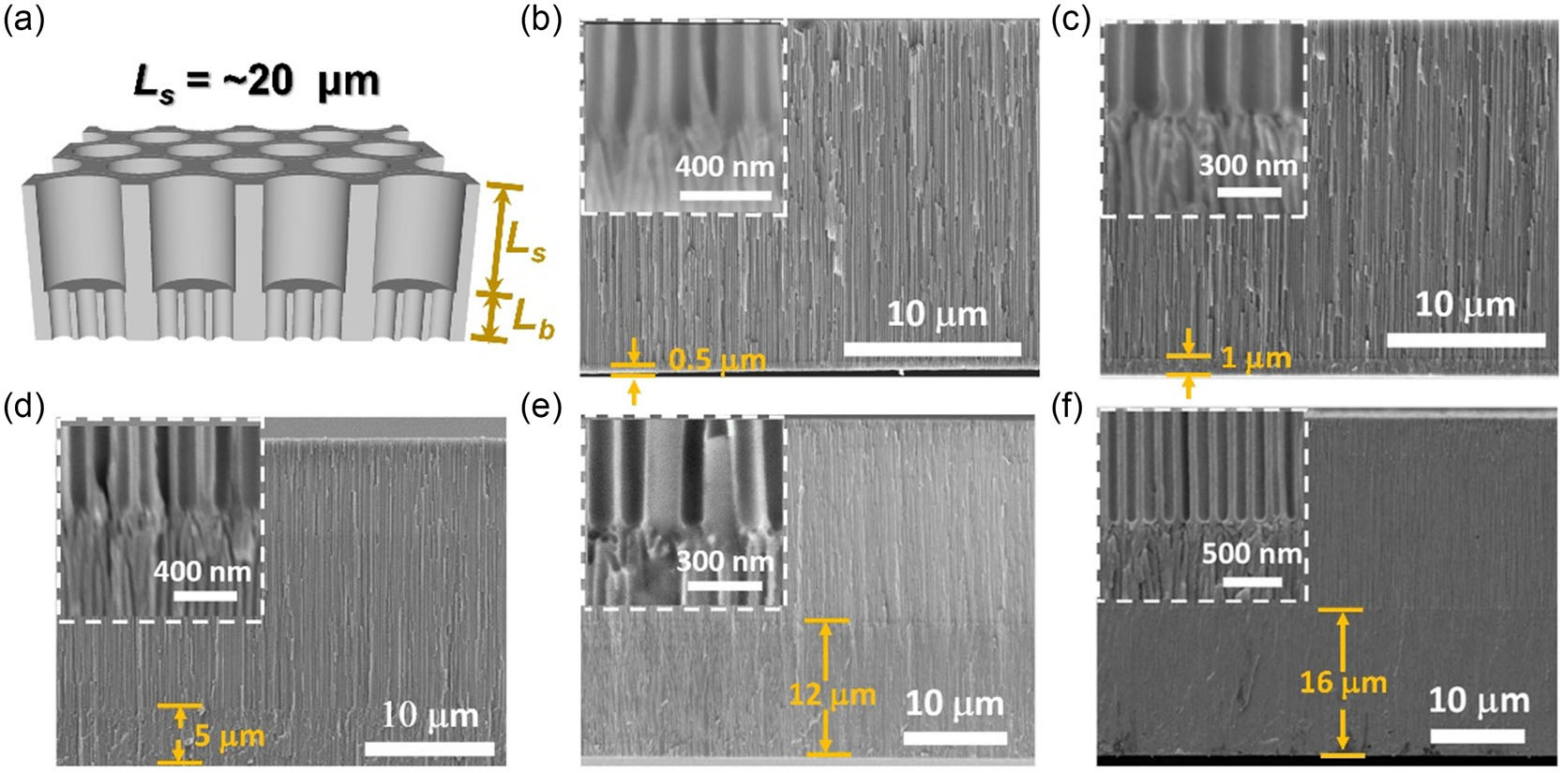
Geometric characterization of the fabricated BAN membranes. a) Schematic of the BAN membrane comprising large stem channels and small branch channels. b–f) Cross‐sectional SEM images of the BAN membranes with *L*_b_ of about (b) 0.5 μm, (c) 1 μm, (d), 5 μm, (e) 12 μm, and (f) 16 μm. *L*_s_ is controlled at ≈20 μm. Insets depict the magnified images of the junctions between stem and branch nanochannels.

All the BAN membranes studied were fabricated via the modified two‐step anodization processes, modified from our previous work^[^ ^36^ ^]^ (Figure S1, Supporting Information). As shown in **Figure** **2** and S2 and S3 (Supporting Information), the BAN membrane is composed of two interconnected nanochannel regions, a region of large‐sized stem channels of length *L*_s_, and a region of small‐sized branched channels of length *L*_b_. Moreover, the fabricated nanochannels in BAN membranes are with highly ordered and aligned pore structures, which are advantageous to ion transportation and osmotic energy generation. The scanning electron microscope (SEM) images shown in Figure S2 and S3 (Supporting Information) revealed that the pore diameters of stem and branched channels in all BAN membranes were controlled at about 105 ± 5 and 26 ± 2 nm, respectively. The lengths of the stem and branched channels can be tuned by regulating the time spent in the first and second anodization procedures, respectively. For example, we fixed the first anodization time at 1 h and elevated the second anodization time from 0.5 to 7 h, to receive the BAN membranes with stem channel length of ≈20 μm and branched channel lengths increasing from 0.5 to 16 μm (Figure 2 and Table S1, Supporting Information). In this study, we named these BAN membranes with branched channel lengths of about 0.5, 1, 5, 12, and 16 μm as branch0.5, branch1, branch5, branch12, and branch16, respectively.

### 2.2 Validation of Rectified BAN Membrane

**Figure 3:**
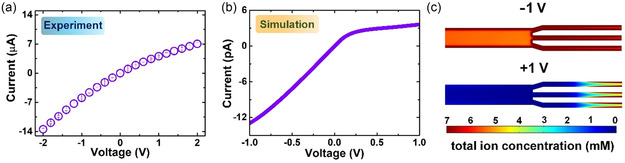
ICR in BAN. a) Illustrated *I–V* curve of the BAN membrane recorded in 1 mm salt solution. b) Simulated *I–V* curve of the BAN system. The parameters used in the charge regulation model include *pK*_a_ = 7, *pK*_b_ = –9.5, *N*_t_ = 1 site nm^−2^, and *C*_salt_ = 1 mm. c) Spatial variations of the total ion concentration near the junction between stem and branch nanochannels for two applied voltages. The ion accumulation and depletion phenomena occur at −1 and +1 V, respectively.

As the next step, we tested ion transport property of the BAN membrane by examining its current–voltage (*I–V*) response using the lab‐made electrochemical cell with two reservoirs filled with the same electrolyte solution.^[^ ^37^, ^38^ ^]^ Experimental result depicted in **Figure** **3**a proved the diode‐like ICR property of the BAN membrane, in which the current at negative voltage is amplified. This behavior can be attributed to the known broken symmetry in pore sizes of stem and branched channels.^[^ ^39^ ^]^

It is known that the isoelectric point (IEP) of AAO is in between 8 and 9.^[^ ^40^, ^41^ ^]^ So, typically the alumina nanochannel in contact with an acid and a neutral electrolyte solutions is positively charged, whereas in the presence of a basic electrolyte solution it becomes negatively charged, and^[^ ^42^ ^]^ so is the BAN membrane. The shift in surface charge of the BAN membrane from positive (acid and neutral) to negative (base) caused the reversal of ICR directions (Figure S4, Supporting Information), similar to the earlier work with the functionalized porous anodic alumina membrane.^[^ ^40^ ^]^

To clearly understand the underlying mechanism behind the ionic rectification of the BAN membrane, we simulated ion transport property of the BAN based on the multi‐ion PNP–NS model.^[^ ^12^, ^35^ ^]^ The applicability of this model has been proven in our previous studies.^[^ ^43^, ^44^ ^]^ The simulated BAN system can be found in Figure S5 (Supporting Information). In contrast to the earlier studies which typically assumed a constant surface charge density on the nanochannel walls,^[^ ^45^, ^46^, ^47^ ^]^ we considered the interfacial chemistry reactions of Al–OH functional groups on the alumina channel walls, $\text{Al}-\text{OH}↔\text{Al}-O^{-}+\;H^{+}$ and $\text{Al}-\text{OH}+\;H^{+}↔\text{Al}-{\text{OH}}_{2}^{+}$,^[^ ^42^ ^]^ in modeling. The mesh‐independent test revealed in Figure S6 (Supporting Information) indicated that the total number of meshes required was ≈1 000 000, to receive convergent and reliable data. The simulated *I–V* curve shown in Figure 3b was in good agreement with the experimental finding (Figure 3a). The ICR behavior can be further supported by the spatial variations of the total ion concentration in the BAN at two applied voltages (Figure 3c). The asymmetry of pore sizes causes the apparent ion enrichment and depletion phenomena near the junction of stem and branched channels at a negative voltage (−1 V) and positive voltage (+1), respectively. The verification of the ionic diode property in the BAN membrane indicates that it is a potential candidate for osmotic energy conversion.

### 2.3 Improved Osmotic Energy Conversion with BAN Membranes

**Figure 4:**
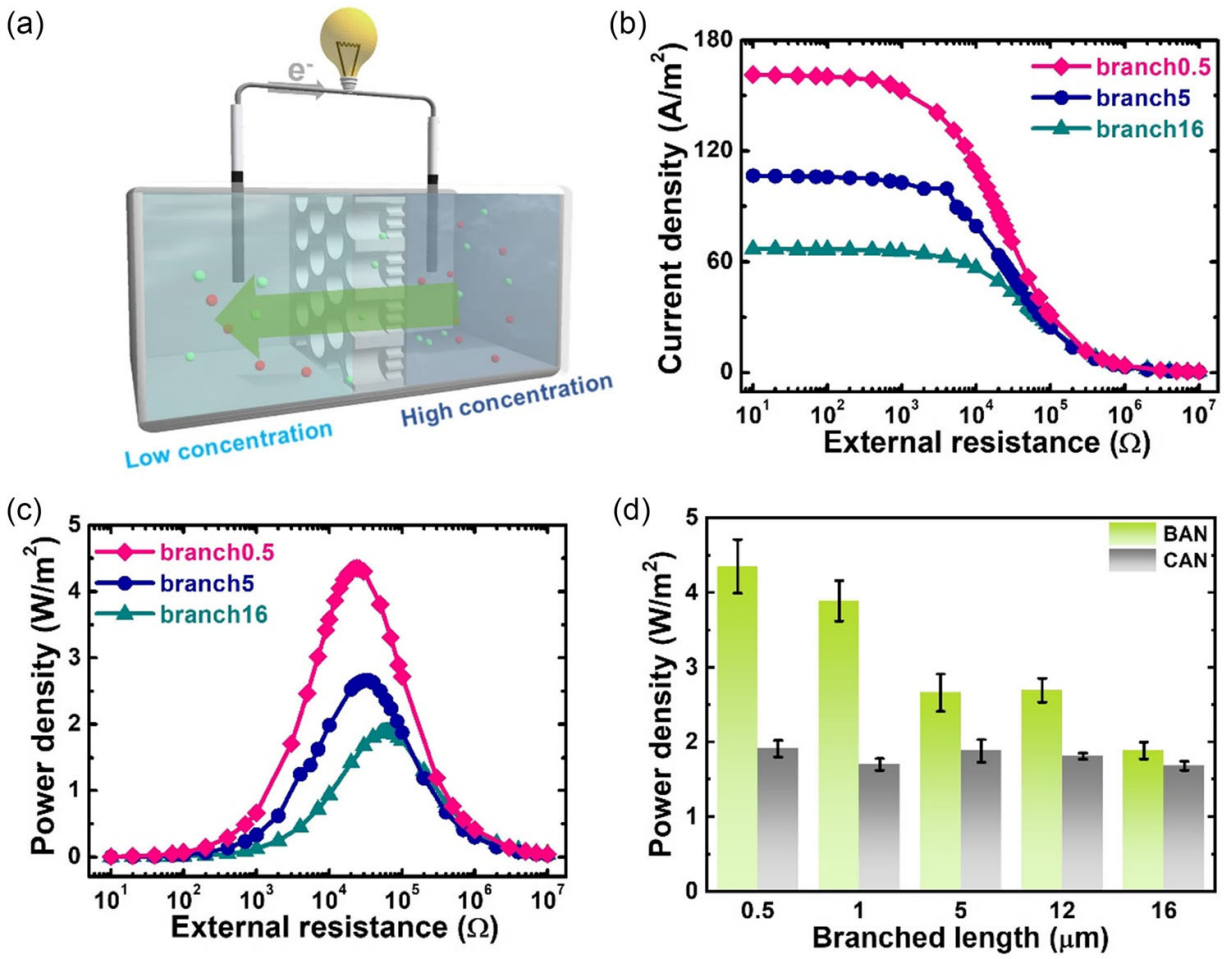
Effect of the branch channel length on osmotic power conversion. a) Schematic of the device used for osmotic energy harvesting. The branch channel side of the BAN membrane was in contact with the higher‐salinity solution. b) Current density and c) power density of BAN membranes with *L*_b_ of about 0.5 (branch0.5), 5 (branch5), and 20 μm (branch20) as a function of external resistance under a 500 mm/1 mm NaCl gradient. d) Comparison of the achieved power densities between BAN and CAN membranes. Note that the channel lengths of the CAN membranes are nearly the same as the total lengths of the corresponding BAN membranes.

We then investigated the osmotic energy conversion performance of the above fabricated BAN membranes using the same electrochemical cell in the presence of a 500 mm/1 mm NaCl gradient. Since the BAN membrane can rectify ionic current, we first identified the optimal configuration of the salinity gradient direction for amplification of osmotic energy conversion performance (Figure S7, Supporting Information). When the concentrated solution was placed in the stem‐channel‐side reservoir, the generated open‐circuit voltage (*V*_oc_) and short‐circuit current (*I*_sc_) were about 57.0 mV and 2.20 μA, respectively. If the salinity gradient was reversed, the values of *V*_oc_ and *I*_sc_ were elevated to about 117 mV and 3.20 μA, respectively, indicating the potential of achieving higher osmotic power under this salinity gradient configuration. Thus, we fixed the concentrated solution in the branch‐channel‐side reservoir in the experiments of subsequent osmotic energy conversion performance testing (**Figure** **4**a), which was identified by transferring the harvested osmotic energy to an electrical load resistor located in the external circuit.^[^ ^19^, ^48^ ^]^ The performance of the output power harvested can be calculated by *P* = *I* ^2^ × *R*_L_, where *I* is the measured current and *R*_L_ is the load resistance. For all the tested BAN membranes, the measured current density decreases and the power density goes through a local maximum with the value of *R*_L_ (Figure 4b,c; Figure S8, Supporting Information). The maximum output power density that a BAN membrane can achieve can be attained when its internal resistance is equal to the load resistance.^[^ ^19^ ^]^ As shown in Figure 4c, the branch0.5, branch5, and branch20 BAN membranes can achieve the maximum power densities of about 4.35, 2.67, and 1.89 W m^−2^ at the load resistance of 24, 32, and 62 kΩ, respectively. The increase of the length of branched channels leads to the increase of membrane resistance and thus a decrease in osmotic power performance.

To demonstrate the enhanced osmotic energy harvesting performance with the proposed BAN membranes, we further compared their achieved performances with the corresponding cylindrical alumina nanochannel (CAN) membranes having similar thicknesses (Figure 4d; Figure S9 and S10, Supporting Information). Different from the asymmetric BAN membranes, the CAN membranes are composed of uniform and symmetric channels (Figure S9, Supporting Information). Figure 4d clearly shows that the power densities outputted by the BAN membranes all significantly surpassed those by the conventional CAN membranes of similar thicknesses. For example, as compared to the corresponding CAN membrane, the branch0.5 BAN membrane promotes the osmotic power by ≈130% (from ≈1.91 to ≈4.35 W m^−2^). Besides, both the current and power densities of the BAN membrane consistently outperformed those generated by the CAN membranes having the smaller pore size of ≈25 nm and larger pore size of ≈105 nm (Figure S11, Supporting Information). These findings further highlight the significance of a dual attribute, emphasizing that achieving high‐performance osmotic energy generation relies not solely on high ion selectivity or high ESR alone. In particular, Figure S12 (Supporting Information) further shows that the membranes with the composition of smaller channels (branch0.5 and CAN20_25 nm) can keep higher ion selectivity, evident in their higher *V*_OC_ compared to the corresponding CAN membrane with larger channels (CAN20_105 nm).^[^ ^4^ ^]^ On the other hand, the membranes with the composition of larger channels (branch0.5 and CAN20_105 nm) can exhibit faster ion transport (larger *I*_SC_) than the corresponding CAN membrane with smaller channels (CAN20_25 nm), possibly stemming from the enhanced ESR values. Therefore, by improving both ion selectivity and ESR, and ascribed to the induced ICR property, the BAN membrane proves to be an ideal system for osmotic energy generation.

**Figure 5:**
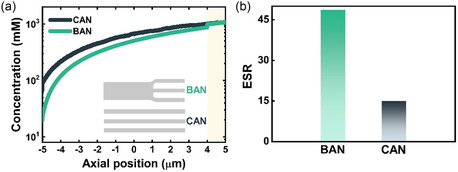
a) Axial variations of the cross‐sectionally averaged total ion concentration in the BAN and CAN systems under a 500 mm/1 mm concentration gradient. The sizes of the stem and branch channels of BAN are set at 100 and 25 nm, respectively. The channel size of CAN is set at 25 nm. Yellow region highlights the interior of branch channels in the BAN system. The insets illustrate the simulated systems (not to the actual scale). b) Calculated ESR values across the BAN and CAN systems.

The significantly enhanced osmotic energy performance of the BAN membranes can be intuitively ascribed to their asymmetric pore structures of stem and branched channels, which induce ICR property and diminish the ICP effect. The former has been confirmed in Figure 3 and the latter can be further demonstrated in **Figure** **5**, where we simulated osmotic ion transport properties in both the BAN and CAN systems having the same total channel length of 10 μm. As compared to the bulk concentration applied (1 mm), the total ion concentration at the low‐concentration entrance of the CAN system is appreciably promoted (Figure 5a), due to the strong ICP effect in symmetric ion‐selective channels. This leads to a significant reduction of the ESR to 14.9‐fold (Figure 5b), as compared to a 500‐fold BSR value applied. On the other hand, Figure 5a also shows that the designed asymmetric pore structures of large‐sized stem and small‐sized branched channels in the BAN system are indeed able to undermine the ICP effect. The significantly decreased total ion concentration at the low‐concentration entrance of the BAN leads to an increase of the ESR to 48.7‐fold (Figure 5b) and hence a more effective osmotic energy conversion than that of the CAN.

### 2.4 Maximizing Osmotic Energy Conversion

**Figure 6:**
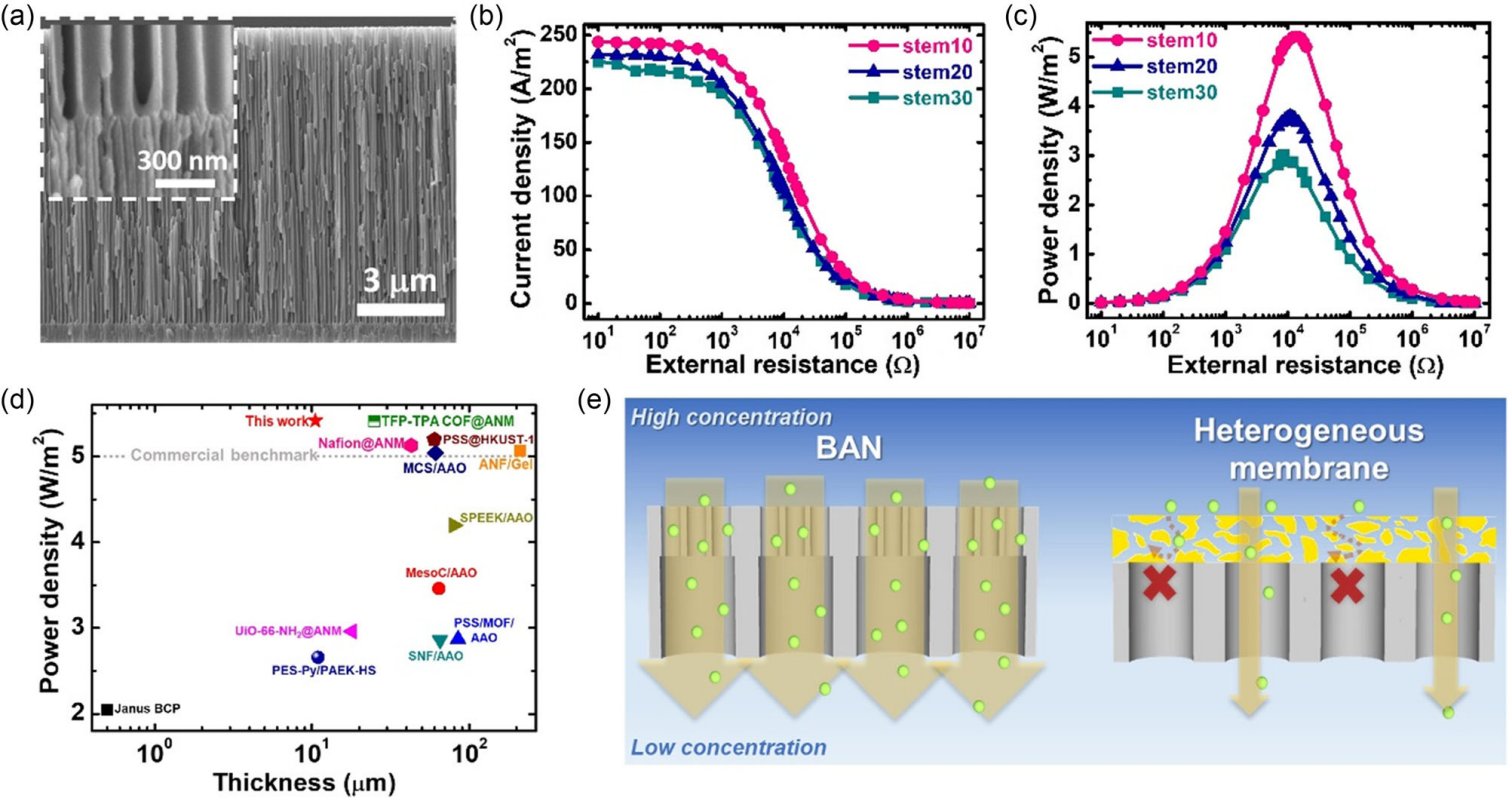
Effect of the stem channel length on osmotic power conversion. a) SEM images of the stem10 BAN membrane (stem channel length of ≈10 μm and branch channel length of ≈0.6 μm). Inset depicts the magnified image of the junction between stem and branch nanochannels. b) Current density and c) power density of the BAN membranes with *L*_s_ of about 10 (stem10), 20 (stem20), and 30 μm (stem30) as a function of external resistance under a 500 mm/10 mm NaCl gradient. All the BAN membranes used have nearly the same *L*_b_ between 0.5 and 0.6 μm. d) Comparison of the achieved osmotic power performance between the fabricated BAN membrane and the state‐of‐the‐art ion‐selective membranes reported previously. e) Schematic of the BAN membrane featuring highly ordered and interconnected pore structures, leading to reduced interfacial resistance and improved ionic flux compared to typical heterogeneous membranes.

In the final session, we tried to maximize the osmotic energy harvesting performance of the BAN membranes by changing the length of the stem channels. Therefore, we prepared three independent BAN membranes with fixed branched channel length of ≈0.5–0.6 μm and various stem channel lengths of 10, 20, and 30 μm (named as stem10, stem20, and stem30). The fabrication parameters and pore geometries of these BAN membranes can be found in Table S2 (Supporting Information). The SEM images shown in **Figure** **6**a prove the successful fabrication of the stem10 BAN membrane. Note that we were unable to fabricate the distinct thinner BAN membrane than the stem10. To mimic the mixing of artificial seawater and river water, we examined the osmotic energy conversion performance of these BAN membranes in a fixed 500 mm/10 mm NaCl gradient at neutral pH and room temperature (Figure 6b,c), the same as that employed in previous studies with heterogeneous membranes.^[^ ^16^, ^17^, ^18^, ^19^, ^20^, ^21^, ^22^, ^23^, ^24^, ^25^, ^26^, ^27^ ^]^ The achieved pore densities for the stem10, stem20, and stem30 BAN membranes were about 5.42, 3.76, and 3.01 W m^−2^, respectively (Figure 6c). The reduction in the stem channel length improves the osmotic energy generation of the BAN membrane due to the facilitated osmotic ion transport.^[^ ^49^, ^50^ ^]^ It is noteworthy that through the optimal design, the BAN membrane achieves high‐performance level for harvesting osmotic energy, and the produced power density of 5.42 W m^−2^ satisfies the commercial demand (5 W m^−2^) and surpasses most of the state‐of‐the‐art heterogeneous membranes^[^ ^16^, ^17^, ^18^, ^19^, ^20^, ^21^, ^22^, ^23^, ^24^, ^25^, ^26^, ^27^ ^]^ (Figure 6d; Table S3, Supporting Information). The highly efficient osmotic energy harvesting of the BAN membrane can be attributed to its important feature of interconnected, ordered, and aligned asymmetric stem and branched nanochannels, which can not only induce ICR property but also significantly promote ESR. Additionally, the interconnected nature between the branched and stem channels could lead to a lower interfacial resistance compared to a conventional heterogeneous membrane, which offers longer transport pathways (Figure 6e).

## 3 Conclusion

In summary, the BAN membranes consisting of interconnected stem channels and branched channels were successfully engineered. Both experimental and simulation results demonstrated the ICR behavior of the fabricated membranes, which is attributed to the structural asymmetry of the interconnected pores. Besides, the BAN membranes were shown to outperform their symmetrical counterparts (i.e., CAN) in terms of output power density, which was further supported by the significantly higher ESR of the former. The effects of branch and stem lengths on osmotic power generation were also investigated. Expectedly, a longer branch/stem length results in a lower output power. Among all the lengths tested, branch and stem channel lengths of ≈0.5–0.6 and 10 μm, respectively, produced the highest power density of 5.42 W m^−2^, exceeding the commercial benchmark (5 W m^−2^) and that of most of the heterogeneous membranes reported previously. This enhancement in osmotic power generation can also be ascribed to the orderly aligned and interconnected open pore structure of the BAN, which reduces interfacial resistance of the heterogeneous membrane. This work paves a new way for the development of novel high‐performance osmotic power generators.

## 4 Experimental Section

4.1

4.1.1

##### Fabrication of BAN Membranes

All the BAN membranes used were fabricated through the two‐step anodization processes, modified from our previous work^[^ ^36^ ^]^ (Figure S1, Supporting Information). In brief, high‐purity aluminum sheets (Al, 99.9995%, Strem Chemicals) were electropolished in a mixed solution of perchloric acid and 95% ethanol (1:4 in volume ratio) under vigorous stirring to obtain the mirror‐like surface. Preanodization was performed in 0.3 m oxalic acid (H_2_C_2_O_4_) under 60 V for 30 min, in which the temperature was well controlled at 20 °C by a circulating system. After preanodization, the oxide layer with disordered pore structures was then removed using phosphoric acid/chromic acid solution at 60 °C for 90 min, to obtain highly ordered arrays of hemispherical Al concaves. Then the first anodization process was performed under the same anodization condition as the preanodization and the length of stem channels can be regulated by tuning the first anodization time. The highly aligned and large‐sized stem channels were obtained through a pore widening process using 5 wt% phosphoric acid (H_3_PO_4_) at 30 °C for 50 min. In the second anodization, the small‐sized branch channels could fabricated by reducing the anodization voltage to 25 V in 0.3 m sulfuric acid (H_2_SO_4_). The BAN membranes with various branched channel lengths could fabricated by modulating the second anodization time. To prepare a free‐standing BAN membrane, the residual aluminum substrate was removed using a mixed solution of copper dichloride and chloric acid and finally the barrier alumina layer was etched using 5 wt% H_3_PO_4_ at 30 °C for 26 min. The pore geometry of BAN membranes was characterized using field emission scanning electron microscope (JEOL, JSM‐7900F).

##### Fabrication of CAN Membranes

All the CAN membranes used were fabricated using the similar two‐step anodization processes with 0.3 m H_2_SO_4_.^[^ ^51^ ^]^ The anodization voltage was 25 V and the temperature was fixed at 1 °C. The total channel length (or thickness) of CAN membranes were well controlled by regulating the second anodization time from 4.4 to 8.5 h.^[^ ^42^ ^]^

##### Electrical Measurement

Ion transport property and osmotic energy conversion performance of the fabricated membranes were tested using a lab‐made electrochemical cell. All measurements of current–voltage curves were conducted with a Keithley 6487 picoammeter/voltage source (Keithley Instruments) at room temperature, with the testing membrane area of 0.03 mm^2^, through a pair of Ag/AgCl electrodes. The working electrode was set at the branched channel side and the reference electrode was fixed at the stem channel side. The pH value was set to be neutral (≈pH 6) unless stated otherwise. The actual output performance of harvested osmotic energy was estimated by introducing a concentration gradient of NaCl solutions and connecting the power source with a tunable resistance box (RS‐200, IET Labs Inc.), which was identical to the measurement setup in previous studies.^[^ ^19^, ^22^, ^28^ ^]^

##### Theoretical Simulation

Ion transport properties of the BAN and CAN systems in the absence and presence of a NaCl gradient were simulated by the multi‐ion PNP–NS model together with considering the surface chemistry reactions of Al–OH functional groups on the alumina nanochannel walls^[^ ^12^ ^]^ (see details in Supporting Information). Thus, the surface charge density of alumina nanochannels can be described by^[^ ^35^ ^]^

(1) $$\sigma_{w}=-(FN_{w})\left\{\frac{10^{-{\text{pK}}_{a}}-10^{-{\text{pK}}_{b}}{\left({\left[H^{+}\right]}_{w}\right)}^{2}}{10^{-{\text{pK}}_{a}}+10^{-{\text{pK}}_{b}}{\left({\left[H^{+}\right]}_{w}\right)}^{2}+{\left[H^{+}\right]}_{w}}\right\}$$

where *pK*_a_ = −log*K*_a_ and *pK*_a_ = −log*K*_b_ with *K*_a_ and *K*_b_ being the equilibrium constants of the deprotonation and protonation reactions of the Al–OH functional groups on the channel wall, respectively. *F* is the Faraday constant, *N*_w_ is the total site density of Al–OH functional groups on the channel wall, and [H^+^]_w_ is the surface molar concentration of protons on the channel wall. The commercial finite element software COMSOL Multiphysics 4.3a was used. To receive affordable computational cost, the simulated BAN system was built on a single BAN, consisting of a stem channel and three branched channels, connected to two large identical reservoirs (Figure S4, Supporting Information). In the simulation, we assumed *N*_w_ = 1 site nm^−2^, *pK*_a_ = 7, and *pK*_b_ = −9.5, corresponding to the IEP of 8.25, which lay in the typical values (e.g., 8–9) for the alumina surface.^[^ ^40^, ^41^ ^]^ Other details of the model can be found in the Supporting Information.

##### Statistical Analysis

Achieved power density data of the BAN and CAN membranes were obtained over three replicates and expressed as mean ± standard deviation (*n* = 3). The presented values were computed by Excel and plotted by OriginPro 2018.

## Conflict of Interest

The authors declare no conflict of interest.

## Supporting information

Supplementary Material

## Data Availability

The data that support the findings of this study are available from the corresponding author upon reasonable request.
