# Supplementary material for: Understanding the Enhanced Osmotic Energy Conversion in Heterogeneous Membranes Using Engineered Branched Alumina Nanochannel Membranes
Source: Small Sci. 2023 Nov 27;4(1):2300167. doi: 10.1002/smsc.202300167 (PMC11935025; doi:10.1002/smsc.202300167)
Supplement: Supplementary file 1 — Supplementary Material [file SMSC-4-2300167-s001.pdf]

Supporting Information for

Understanding the Enhanced Osmotic Energy Conversion in Heterogeneous Membranes Using Engineered Branched Alumina Nanochannel Membranes

Yen-Shao Su,[1,+] *Amalia Rizki Fauziah,*[1,+] *Chung-Yi Wong,*[1,+] *Ting-Yi Huang,*[1]

Li-Hsien Yeh[1,2,*]

[1]Department of Chemical Engineering, National Taiwan University of Science and Technology, Taipei 10607, Taiwan

[2]Advanced Manufacturing Research Center, National Taiwan University of Science and Technology, Taipei 10607, Taiwan

+ These authors contributed equally to this work.

[*] Corresponding author:

Email: lhyeh@mail.ntust.edu.tw (Li-Hsien Yeh)

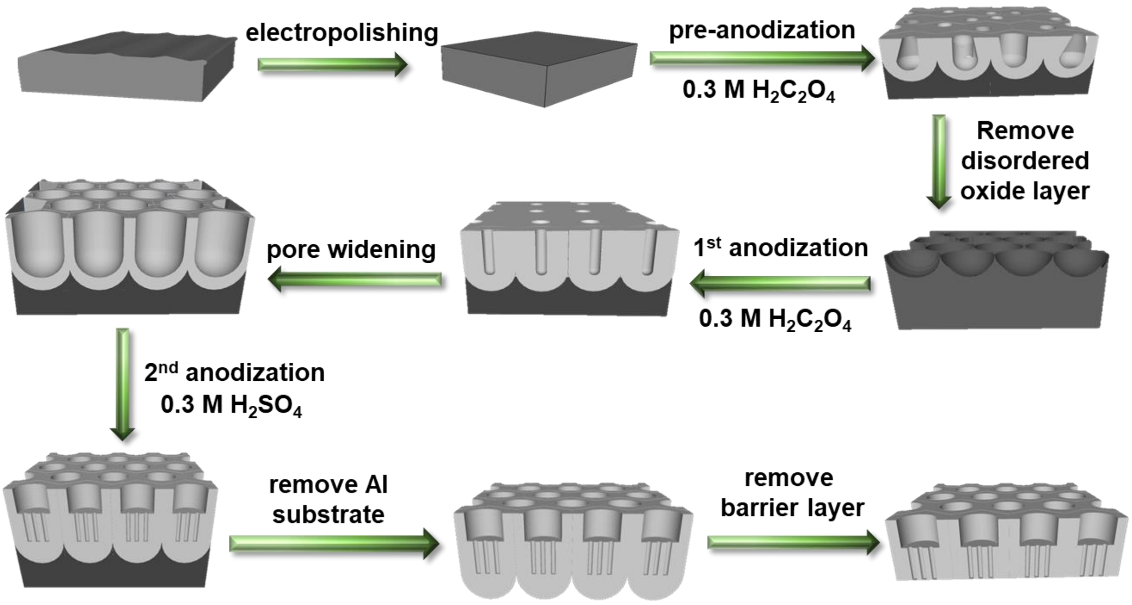

Figure S1. Schematic of the modified two-step anodization for fabricating branched alumina nanochannel (BAN) membranes comprising large stem channels and small branch channels. The oxalic acid ($H_2C_2O_4$) was used in the pre-anodization and 1st anodization, and sulfuric acid (H_2SO_4) was used in the 2nd-anodization.

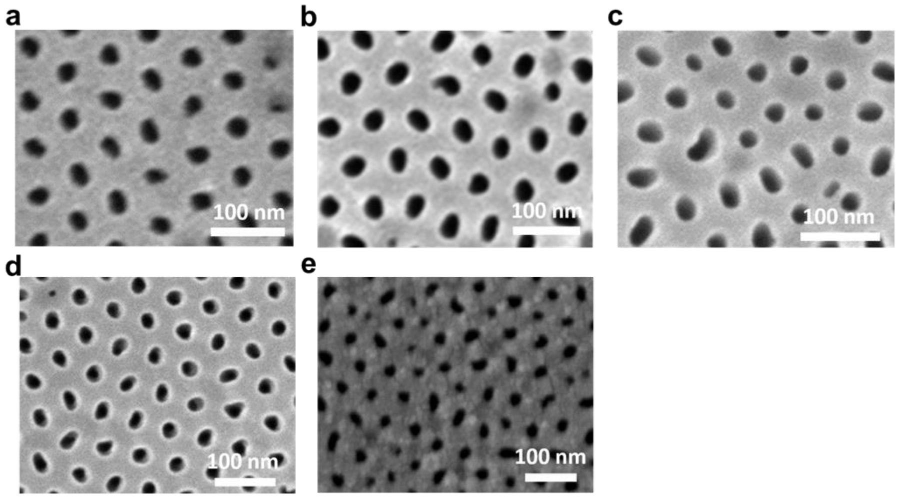

Figure S2. SEM images of the branch channels of the BAN membranes considered in Figures 2b-f with the corresponding branch channel length of about (a) 0.5 μm (branch0.5), (b) 1 μm (branch1), (c), 5 μm (branch5), (d) 12 μm (branch12), and (e) 16 μm (branch16). The diameters of branch channels in these BAN membranes are *ca.* 26±2 nm.

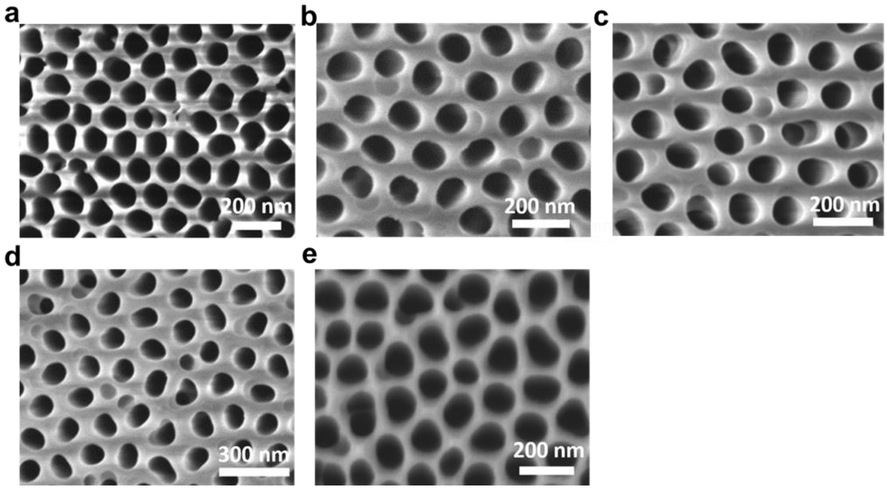

Figure S3. SEM images of the stem channels of the BAN membranes considered in Figure 2b-f with the corresponding branch channel length of about (a) 0.5 μm (branch0.5), (b) 1 μm (branch1), (c), 5 μm (branch5), (d) 12 μm (branch12), and (e) 16 μm (branch16). The diameters of stem channels in these BAN membranes are *ca.* 105±5 nm.

Table S1. Geometric characterizations of BAN membranes with tunable branched channel lengths estimated from SEM.

	**branch0.5**	**branch1**	**branch5**	**branch12**	**branch16**
**1st anodization time (hr)**	1	1	1	1	1
**2nd anodization time (hr)**	0.5	1	3	5	7
D_s **(nm)**	~105	~105	~105	~105	~105
D_b **(nm)**	~26	~26	~26	~26	~26
L_s **(μm)**	~20	~21	~20	~20	~21
L_b **(μm)**	~0.5	~1.0	~5.0	~12	~16
**Total length (μm)**	~20.5	~22	~25	~32	~37

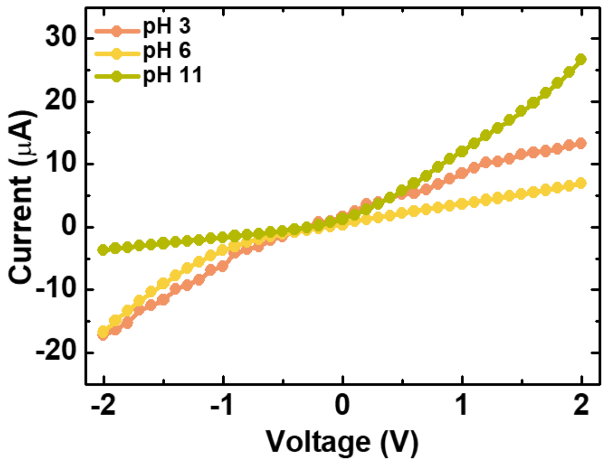

Figure S4. The pH effect on ion current rectification properties of an independently prepared BAN membrane recorded in 1 mM KCl solution.

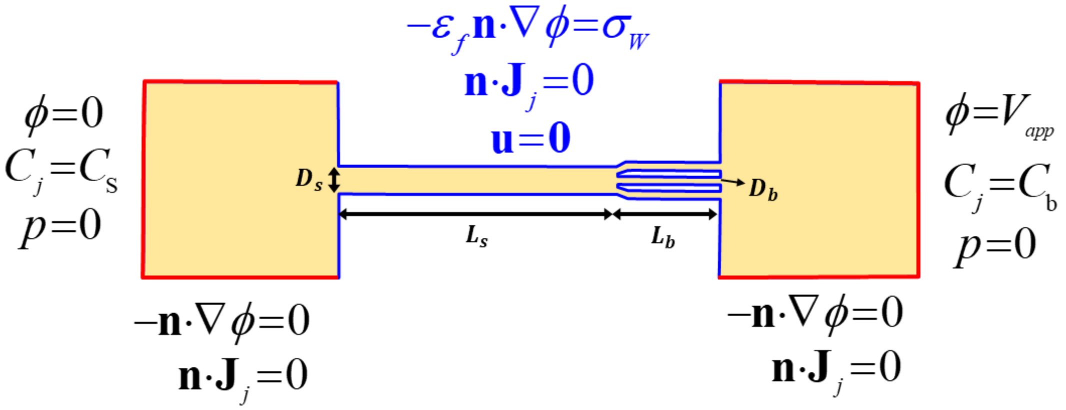

Figure S5. Simulation system and the corresponding boundary conditions for the considered BAN system. The stem (D_s) and branch (D_b) channels were fixed at 100 nm and 25 nm, respectively. The stem channel length (L_s) is 9 µm and the branch channel length (L_b) is 1 µm.

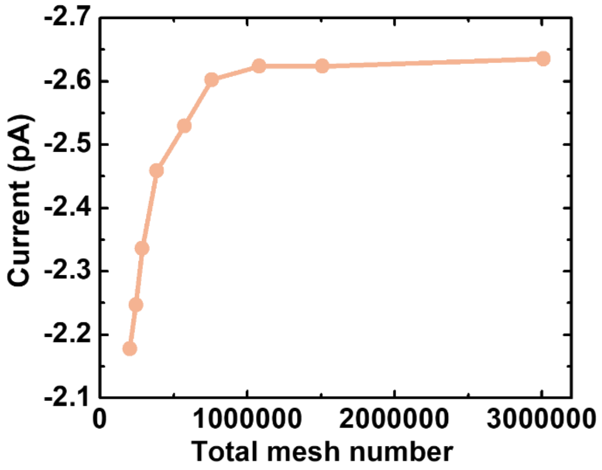

Figure S6. The mesh-independent test for the considered BAN system conducted at 1 mM KCl and -0.1 V. Typically, the total number of meshes (>1,000,000) is required to achieve the mesh-independence, validating the credibility of the simulation results.

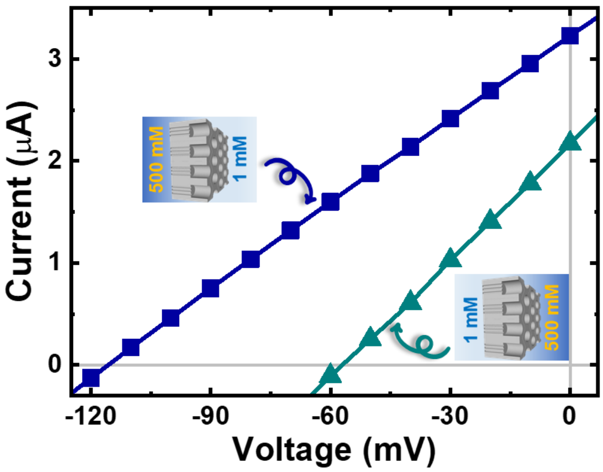

Figure S7. Illustrated I-V curves of a BAN membrane tested in 500-fold NaCl gradient under two configurations of opposite salinity gradients.

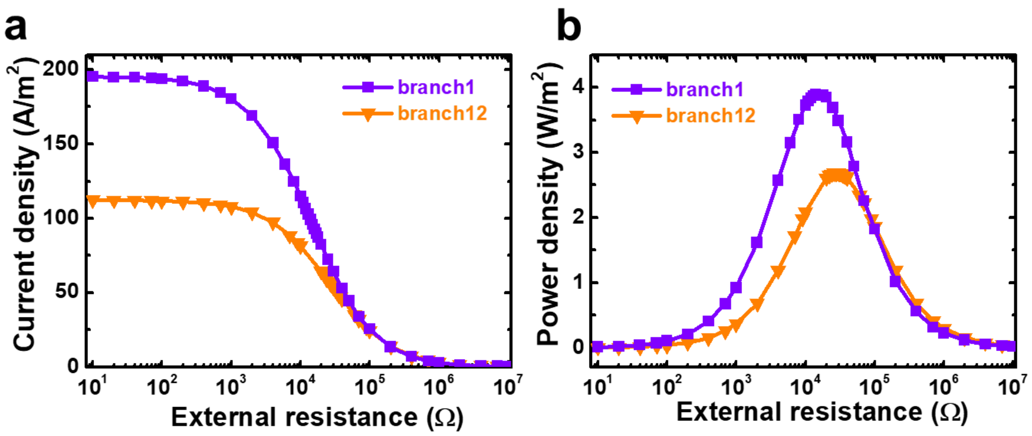

Figure S8. (a) Current density and (b) power density of the BAN membranes with branch channel lengths of 1 μm (branch1) and 12 μm (branch12) as a function of external resistance under a 500 mM/1 mM NaCl gradient.

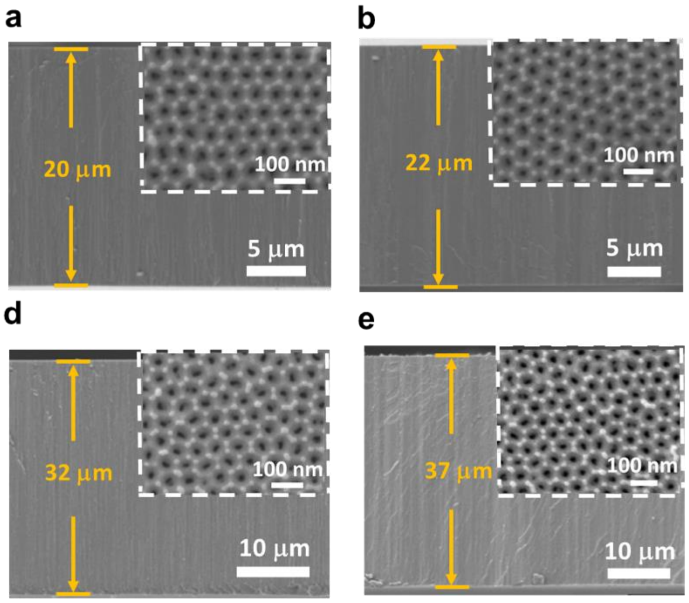

Figure S9. SEM images of the CAN membranes considered with total channel lengths of (a) 20 μm (CAN20), (b) 22 μm (CAN22), (c) 25 μm (CAN25), (d) 32 μm (CAN32), and (e) 37 μm (CAN37). Insets depict that all the CAN membranes have pore diameters about 24±2 nm.

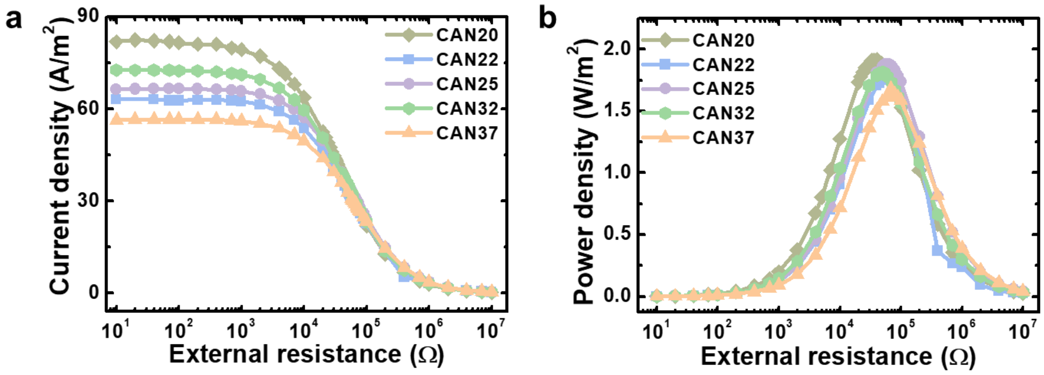

Figure S10. (a) Current density and (b) power density of the CAN membranes with total channel lengths of 20 μm (CAN20), 22 μm (CAN22), 25 μm (CAN25), 32 μm (CAN32), and 37 μm (CAN37) as a function of external resistance under a 500 mM/1 mM NaCl gradient. The maximum power densities for CAN20, CAN22, CAN25, CAN32, and CAN37 were about 1.91, 1.70, 1.88, 1.81, and 1.68 W/m^2, respectively.

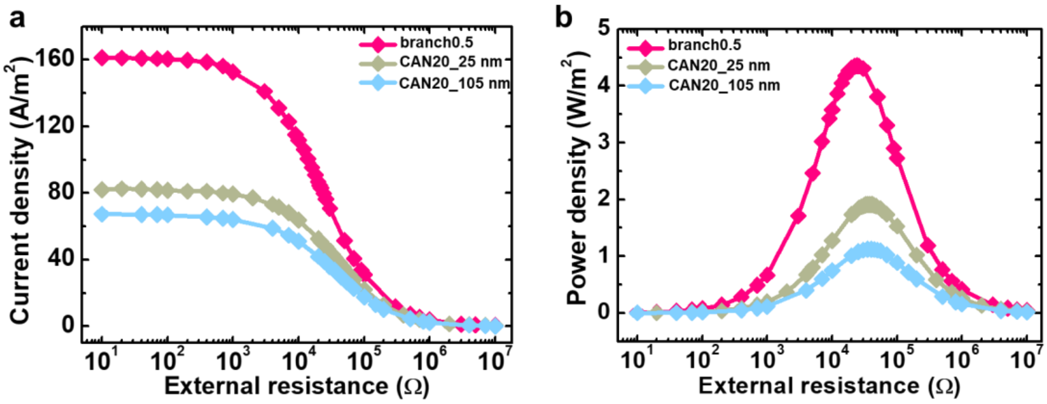

Figure S11. The outputted (a) current density and (b) power density from the BAN membrane (branch05) in comparison with those from the CAN membranes with different pore sizes of 25 nm (CAN20_25 nm) and 105 nm (CAN20_105 nm) under a 500 mM/1 mM NaCl gradient. The maximum power densities achieved were about 4.34, 1.91, and 1.23 W/m^2 for branch0.5, CAN20_25 nm, and CAN20_105 nm, respectively.

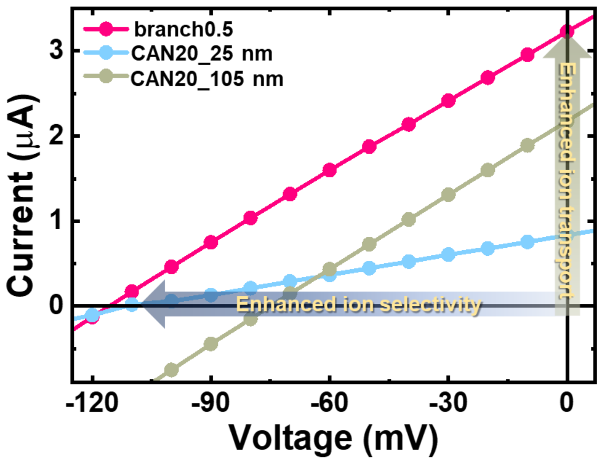

Figure S12. A comparison of the I-V curves of the BAN membrane (branch0.5) and the two corresponding CAN membranes with two distinct pore sizes (CAN20_25 nm and CAN20_105 nm) under a 500 mM/1 mM NaCl gradient. Typically, the larger the V_{OC} the larger the ion selectivity, and the larger the I_{SC} the faster the ion transport in the nanofluidic membrane. The membranes with the composition of smaller channels (branch0.5 and CAN20_25 nm) can hold higher ion selectivity, while the membranes with the composition of larger channels (branch0.5 and CAN20_105 nm) can realize faster ion transport due to the larger ESR. In addition to the high ion selectivity and large ESR, the rectification property of the BAN membrane renders it with the highest osmotic energy conversion performance.

Table S2. Geometric characterizations of BAN membranes with tunable stem channel lengths estimated from SEM.

	**stem10**	**stem20**	**stem30**
**1st anodization time (hr)**	0.5	1	1.5
**2nd anodization time (hr)**	0.3	0.5	0.75
L_s **(μm)**	~10	~20	~30
L_b **(μm)**	~0.6	~0.5	~0.5
D_s **(nm)**	~105	~105	~105
D_b **(nm)**	~26	~26	~26

Table S3. Comparison of the osmotic power density achieved by the present BAN membrane with that by the previously reported heterogeneous membranes. All the data was estimated at neutral pH and room temperature.

Heterogeneous membrane	Thickness (μm)	NaCl gradient (mM/mM)	Power density (W/m^2)	Ref.
Janus BCP	0.5	500/10	2.04	[1]
MesoC/AAO	64.2	500/10	3.46	[2]
PSS/MOF/AAO	85	500/10	2.87	[3]
SNF/AAO	65	500/10	2.86	[4]
UiO-66-NH_2@ANM	18	500/10	2.96	[5]
SPEEK/AAO	80	500/10	4.2	[6]
MCS/AAO	61	500/10	5.04	[7]
PSS/HKUST-1	60	500/10	5.2	[8]
Nafion@ANM	42.9	500/10	5.13	[9]
TFP-TPA COF@ANM	25	500/10	5.41	[10]
PES-Py/PAEK-HS	11	500/10	2.66	[11]
ANF/Gel	212	500/10	5.06	[12]
**BAN membrane**	**10.5**	**500/10**	**5.42**	**This work**

Theoretical Modeling

The modeling BAN system considered is depicted in Figure S5, where a BAN, consisting of a main large stem channel and three small branched channels, is connected to two large and identical reservoirs. In order to avoid the reservoir effect on ion transport at nano-confinement, we assumed that the width and length of the two reservoirs are 2000 nm.[13] The pore diameter and length of the stem channel are D_{s} and L_{s}, respectively, and D_{b} and L_{b} are the pore diameter and length of branched channels. To reveal the actual experimental setup in model system, the ionic concentrations at the stem channel side is considered as C_S and the voltage is grounded ($\phi = 0$), while those at the branched channel side is C_b and a applied voltage is applied, i.e., $\phi = V_{\mathrm{app}}$.

Ion transport properties in the considered nanofluidic systems (BAN and CAN systems) can be described by the following coupled multi-ion Poisson-Nernst-Planck and Navier-Stokes (PNP-NS) model,[14, 15]

$$-\varepsilon_f \nabla^2 \phi = \rho_e = \sum_{j=1}^{4} F z_j C_j \tag{S1}$$

$$\nabla \cdot \mathbf{J}_j = \nabla \cdot (\mathbf{u} C_j - D_j \nabla C_j - D_j \frac{z_j F C_j}{RT} \nabla \phi) = 0,\ j = 1, 2, 3, 4 \tag{S2}$$

$$\mu \nabla^2 \mathbf{u} - \nabla p - \rho_e \nabla \phi = 0 \tag{S3}$$

$$\nabla \cdot \mathbf{u} = 0 \tag{S4}$$

For the above equations, ϕ is the electrical potential; ρ_e is the space charge density of mobile ions; ε_f and F are fluid permittivity and Faraday constant, respectively; z_j, C_j, $\mathbf{J}_j$, and D_j are the valence, concentration, flux, and diffusivity of ionic species j (j=1 for K^+ or Na^+, j=2 for Cl^-, j=3 for H^+, and j=4 for OH^-), respectively; μ, R, and T are the fluid viscosity, gas constant, and absolute fluid temperature, respectively; $\mathbf{u}$ and p are the

fluid velocity and hydrodynamic pressure, respectively.

Moreover, we considered the actual interfacial protonation and deprotonation equilibrium reactions of alumina hydroxyl groups on the BAN walls[16] in the modeling,

$$\mathrm{Al-OH+H^{+} \leftrightarrow Al-OH_2^{+}} \tag{S5}$$

$$\mathrm{Al-OH \leftrightarrow Al-O^{-} + H^{+}} \tag{S6}$$

Let K_b and K_a be the equilibrium constants of these two interfacial reactions. Thus, the surface charge density (σ_w) of the BAN can be described as[14]

$$\sigma_w = -(FN_w)\left\{\frac{10^{-\mathrm{p}K_a} - 10^{-\mathrm{p}K_b}\left([\mathrm{H}^+]_w\right)^2}{10^{-\mathrm{p}K_a} + 10^{-\mathrm{p}K_b}\left([\mathrm{H}^+]_w\right)^2 + [\mathrm{H}^+]_w}\right\} \tag{S7}$$

where $\mathrm{p}K_a = -\log K_a$ and $\mathrm{p}K_b = -\log K_b$. F is the Faraday constant, N_w is the total site density of Al-OH functional groups on the BAN wall, and $[\mathrm{H}^+]_w$ is the surface molar concentration of protons on the BAN wall. In the modeling, we assumed $N_w = 1\ \mathrm{site/nm^2}$, $\mathrm{p}K_a = 7$, and $\mathrm{p}K_b = -9.5$, corresponding to the IEP of 8.25, which lies in the typical values (*e.g.*, 8-9) for the alumina surface.[17, 18] Other details of the boundary conditions can be found in our previous papers.[15, 16]

The ionic current through the BAN system can be calculated by

$$I_{\mathrm{channel}} = \int_S F\left(\sum_{j=1}^{4} z_j \mathbf{J}_j\right)\cdot \mathbf{n}\, dS\,, \tag{S8}$$

where **n** is unit normal vector and *S* denotes either end of two reservoirs.

References

[1] Zhang, Z.; Sui, X.; Li, P.; Xie, G. H.; Kong, X. Y.; Xiao, K.; Gao, L. C.; Wen, L. P.; Jiang, L., *J. Am. Chem. Soc.* **2017**, *139*, 8905-8914.

[2] Gao, J.; Guo, W.; Feng, D.; Wang, H. T.; Zhao, D. Y.; Jiang, L., *J. Am. Chem. Soc.* **2014**, *136*, 12265-12272.

[3] Li, R. R.; Jiang, J. Q.; Liu, Q. Q.; Xie, Z. Q.; Zhai, J., *Nano Energy* **2018**, *53*, 643-649.
[4] Xin, W. W.; Zhang, Z.; Huang, X. D.; Hu, Y. H.; Zhou, T.; Zhu, C. C.; Kong, X. Y.; Jiang, L.; Wen, L. P., *Nat. Commun.* **2019**, *10*, 3876.
[5] Liu, Y. C.; Yeh, L. H.; Zheng, M. J.; Wu, K. C. W., *Sci. Adv.* **2021**, *7*, eabe9924.
[6] Hou, S. H.; Zhang, Q. R.; Zhang, Z.; Kong, X. Y.; Lu, B. Z.; Wen, L. P.; Jiang, L., *Nano Energy* **2021**, *79*, 105509.
[7] Zhou, S.; Xie, L.; Li, X. F.; Huang, Y. A.; Zhang, L. P.; Liang, Q. R.; Yan, M.; Zeng, J.; Qiu, B. L.; Liu, T. Y.; Tang, J. Y.; Wen, L. P.; Jiang, L.; Kong, B., *Angew. Chem.-Int. Edit.* **2021**, *60*, 26167-26176.
[8] Pan, S. F.; Liu, P.; Li, Q.; Zhu, B.; Liu, X. L.; Lao, J. C.; Gao, J.; Jiang, L., *Angew. Chem.-Int. Edit.* **2023**, *62*, e202218129.
[9] Chang, C. W.; Chu, C. W.; Su, Y. S.; Yeh, L. H., *J. Mater. Chem. A* **2022**, *10*, 2867-2875.
[10] Gao, M. Y.; Zheng, M. J.; El-Mahdy, A. F. M.; Chang, C. W.; Su, Y. C.; Hung, W. H.; Kuo, S. W.; Yeh, L. H., *Nano Energy* **2023**, *105*, 108007.
[11] Zhu, X. B.; Hao, J. R.; Bao, B.; Zhou, Y. H.; Zhang, H. B.; Pang, J. H.; Jiang, Z. H.; Jiang, L., *Sci. Adv.* **2018**, *4*, eaau1665.
[12] Zhang, Z.; He, L.; Zhu, C. C.; Qian, Y. C.; Wen, L. P.; Jiang, L., *Nat. Commun.* **2020**, *11*, 875.
[13] Lin, C. Y.; Chen, F.; Yeh, L. H.; Hsu, J. P., *Phys. Chem. Chem. Phys.* **2016**, *18*, 30160-30165.
[14] Yeh, L. H.; Zhang, M.; Qian, S., *Anal. Chem.* **2013**, *85*, 7527-7534.
[15] Yeh, L. H.; Chen, F.; Chiou, Y. T.; Su, Y. S., *Small* **2017**, *13*, 1702691.
[16] Su, Y. S.; Hsu, S. C.; Peng, P. H.; Yang, J. Y.; Gao, M. Y.; Yeh, L. H., *Nano Energy* **2021**, *84*, 105930.
[17] Li, C. Y.; Ma, F. X.; Wu, Z. Q.; Gao, H. L.; Shao, W. T.; Wang, K.; Xia, X. H., *Adv. Funct. Mater.* **2013**, *23*, 3836-3844.
[18] Kosmulski, M., *J. Colloid Interface Sci.* **2009**, *337*, 439-448.
